# Preeclampsia and the Risk of Pancreatitis: A Nationwide, Population-Based Cohort Study

**DOI:** 10.1155/2020/3261542

**Published:** 2020-12-30

**Authors:** Jia-Lun Huang, Wei-Kung Chen, Cheng-Li Lin, Chia-Hung Kao, Hong-Mo Shih

**Affiliations:** ^1^School of Medicine, College of Medicine, China Medical University, Taiwan; ^2^Department of Emergency Medicine, China Medical University Hospital, Taiwan; ^3^Department of Public Health, China Medical University, Taiwan; ^4^Management Office for Health Data, China Medical University Hospital, Taiwan; ^5^College of Medicine, China Medical University, Taiwan; ^6^Graduate Institute of Clinical Medical Science and School of Medicine, College of Medicine, China Medical University, Taiwan; ^7^Department of Nuclear Medicine and PET Center, China Medical University Hospital, Taiwan

## Abstract

**Background:**

Preeclampsia is a multiple organ dysfunction during pregnancy, including hepatic, renal, and neurological dysfunction, and is defined as hypertension and proteinuria occurring after 20 weeks of pregnancy. Clinical features seen in preeclampsia are due to relatively poorly perfused placenta and maternal endothelial dysfunction. Some studies have found that preeclampsia may cause acute pancreatitis due to microvascular abnormalities and visceral ischemia. This retrospective cohort study used the Taiwanese National Health Insurance Research Databases (NHIRD) to study the relationship between preeclampsia and the risk of pancreatitis.

**Methods:**

In total, 606,538 pregnant women were selected from the NHIRD between January 1, 1998 and December 31, 2010. They were divided into a preeclampsia cohort (*n* = 485,211) and a nonpreeclampsia cohort (*n* = 121,327). After adjusting for comorbidities that may induce pancreatitis, we analyzed and compared the incidence of pancreatitis in the two cohorts.

**Results:**

The overall incidence of pancreatitis in the preeclampsia cohort was significantly higher than that in the control cohort (4.29 vs. 2.33 per 10,000 person-years). The adjusted HR of developing pancreatitis increased 1.68-fold (95% CI: 1.19-2.36) in the preeclampsia cohort. In addition, pregnant women with preeclampsia without comorbidities had a significantly high risk of pancreatitis (aHR = 1.83, 95% CI 1.27-2.63). The combined effect of preeclampsia and alcohol-related diseases resulted in the highest risk of pancreatitis (aHR = 43.4, 95% CI: 6.06-311.3).

**Conclusion:**

Compared with patients without preeclampsia, the risk of pancreatitis in patients with preeclampsia is significantly increased after adjusting for demographics and comorbidities. The risk of pancreatitis is greatly increased when preeclampsia is accompanied by alcohol-related diseases, hepatitis C, gallstones, diabetes, or age of 26–35 years. Early identification and effective control of preeclampsia and the associated comorbidities can reduce the risk of pancreatitis and the associated morbidity and mortality.

## 1. Introduction

Preeclampsia is defined as the presence of de novo hypertension (>140 mmHg systolic or >90 mmHg diastolic) after 20 weeks of gestation combined with proteinuria or other maternal organ dysfunction (renal, hepatic, and neurologic diseases) [1–3]. It occurs in 3%-5% of pregnancies worldwide [4]. Risk factors associated with preeclampsia include a past history of preeclampsia, preexisting hypertension, preexisting diabetes, obesity, *multifetal pregnancy*, chronic kidney disease, *advanced maternal age*, antiphospholipid syndrome, and systemic lupus erythematosus [5].

The increased systemic inflammatory response plays a critical role in the pathogenesis of preeclampsia, leading to edema, extravasation, and increased damage to the vascular bed of the placenta, kidneys, and other organs [6]. This leads to poorly perfused placenta and maternal endothelial dysfunction [7]. These changes make preeclampsia a multiorgan syndrome dysfunction, with increased risks of various disorders, including chronic hypertension, diabetes mellitus, ischemic heart disease, cerebrovascular disease, kidney disease, thromboembolism, hypothyroidism, and even memory impairment [8]. Women with preeclampsia have an increased risk of life-threatening obstetric or medical complications. Globally, 10%–15% of maternal deaths due to pregnancy complications are directly related to preeclampsia/eclampsia [9].

Pancreatitis is the inflammation of the pancreatic glandular parenchyma, usually accompanied by abdominal pain and elevated serum pancreatic enzymes. Acute pancreatitis is the most common cause of hospitalization for gastrointestinal diseases in the United States [10]. Several conditions are related to acute pancreatitis, among which gallstones and chronic alcoholism account for approximately two thirds [11]. Systemic inflammatory response syndrome and organ failure are the main causes of death in the first two weeks of acute pancreatitis, and sepsis is the main cause of death after two weeks [12]. In a systematic review of studies of acute pancreatitis, the overall mortality rate is approximately 5% [13].

Severe preeclampsia can cause various systemic abnormalities, such as refractory abdominal pain, impaired liver function, severe hypertension, cerebral or visual disturbances, progressive renal insufficiency, and thrombocytopenia [14, 15]. Some case reports have indicated a link between the development of pancreatic diseases and preeclampsia [16–20], but studies with a higher level of evidence examining this link are lacking. Therefore, this retrospective cohort study explored the relationship between preeclampsia and pancreatitis by using the Taiwanese National Health Insurance Research Database (NHIRD).

## 2. Materials and Methods

### 2.1. Data Source

This retrospective population-based study used data from the Taiwan NHIRD which covers medical benefit claims for over 23 million people (approximately 99% of Taiwan's population) [21]. The NHIRD contains the registry of beneficiaries and all medical service data. These claim files record the disease based on the International Classification of Diseases, Ninth Revision, Clinical Modification (ICD-9-CM). For each beneficiary, a unique encrypted identification number is used to link all insurance information and health care records. This study was approved by the Ethics Review Board of China Medical University, Taichung (CMUH104-REC2-115).

### 2.2. Variables and Participants

We identified all preeclampsia patients from the NHIRD corresponding to the International Classification of Disease, the Ninth Revision (ICD-9) codes 642.4–642.7 from 1998 to 2010. The date of preeclampsia diagnosis was assigned as the index date. For each woman with preeclampsia, we identified a pregnant woman without the history of preeclampsia for the comparison cohort, frequency matched by age (every 5 years span), and pregnancy year. Patients were excluded if they were <18 or >45 years of age and had a history of pancreatitis (ICD-9 code 577) before the index date. The confirmation of pancreatitis events was based on the database from 1998 to 2011 as the study endpoint. All study participants were followed up from the index date to the occurrence of endpoint, death, withdrawal from the database, or the end of 2011, whichever came first. We evaluated several comorbidities, which were defined as the history before the endpoint. They could be related to pancreatitis, namely, alcohol-related diseases (ICD-9 codes 291, 303, 305.00, 305.01, 305.02, 305.03, 790.3, and V11.3), biliary stone (ICD-9 code 574), diabetes mellitus (ICD-9 code 250), hyperlipidemia (ICD-9 code 272), hypertension (ICD-9 codes 401–405), hepatitis B virus (ICD-9 codes V02.61, 070.20, 070.22, 070.30, and 070.32), and hepatitis C virus (ICD-9 codes V02.62, 070.41, 070.44, 070.51, and 070.54).

### 2.3. Statistical Analysis

Distributions of age and comorbidities were compared between cohorts with and without preeclampsia and then tested using the chi-square test for categorical variables and Student's *t-*test for continuous variables. The cumulative incidence curve for pancreatitis was assessed using the Kaplan–Meier method, and intergroup differences were estimated using the log-rank test. We estimated the incidence densities of pancreatitis during follow-up in both cohorts. We evaluated the risk of pancreatitis for preeclampsia patients compared with the comparison cohort by using univariable and multivariable Cox proportional hazards models and presented by hazard ratios (HRs) and corresponding 95% confidence intervals (CIs). Multivariable models were adjusted for age and comorbidities. All statistical analyses were performed using SAS 9.4 (SAS Institute, Cary, NC, USA). We set the significant level at *p* < 0.05 for two-sided testing.

## 3. Results

The eligible study participants consisted of 17 263 patients in the preeclampsia cohort and 69 052 individuals in the nonpreeclampsia cohort. The baseline characteristics of all patients are summarized in Table 1. The mean age of patients with and without preeclampsia was 31.2 ± 5.19 and 31.0 ± 5.22 years, respectively, and 64.0% of the study participants were aged 26–35 years. Compared with patients without preeclampsia, those with preeclampsia were more likely to have biliary stones (1.74%), diabetes mellitus (5.02%), hyperlipidemia (1.81%), hypertension (3.85%), hepatitis B virus (0.74%), and hepatitis C virus (0.27%). The average follow-up duration was 7.03 ± 3.90 years for the preeclampsia cohort and 7.03 ± 3.92 years for the comparison cohort.

**Figure 1:**
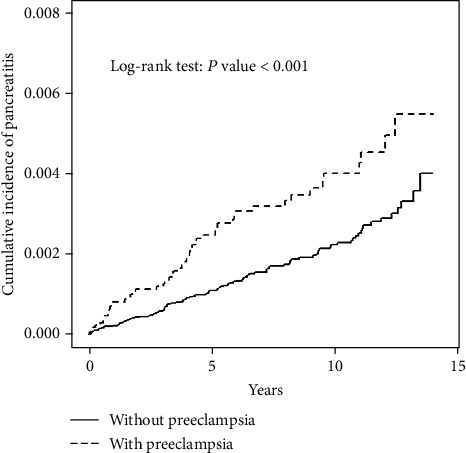
Comparison of cumulative incidence of pancreatitis among patients with preeclampsia and patients in the comparison cohort.

The overall incidence of pancreatitis was greater in the preeclampsia cohort than in the comparison cohort (4.29 vs. 2.33 per 10,000 person-years, crude HR = 1.84, 95% CI 1.32–2.55), and after adjusting for age and comorbidities of alcohol-related disease, biliary stone, diabetes mellitus, hyperlipidemia, hypertension, hepatitis B virus, and hepatitis C virus, the adjusted HR (aHR) was 1.68 (95% CI 1.55–1.78) (Table 2). The pancreatitis incidence increased with age. The risk of pancreatitis was higher in patients with preeclampsia than the comparison cohort for the 26–35 age group (aHR = 2.22, 95% CI 1.43–3.44). Moreover, among patients without comorbidities, the risk of pancreatitis was higher in the preeclampsia cohort than in the comparison cohort (aHR = 1.83, 95% CI 1.27–2.63). Patients with preeclampsia had a higher pancreatitis rate than did the comparison cohort after 14 years of follow-up (log-rank *p* < 0.001, Figure 1).

Table 3 reveals the combined effect of preeclampsia with specific comorbidities. Compared with nonpreeclampsia patients without alcohol-related diseases, a significantly increased risk of pancreatitis was observed in preeclampsia patients with alcohol-related diseases (aHR = 43.4, 95% CI 6.06–311.3), followed by preeclampsia patients without alcohol-related diseases (aHR = 1.80, 95% CI 1.29–2.50) (Table 3). A significantly high pancreatitis risk was observed in patients with both preeclampsia and hepatitis C virus (aHR = 11.0, 95% CI 1.54–78.9) compared with those without either disease. Preeclampsia coexisting with diabetes mellitus (aHR = 5.89, 95% CI 1.87–18.6) or biliary stone (aHR = 3.63, 95% CI 1.59–8.27) was also associated with a high risk of pancreatitis.

## 4. Discussion

This is the first nationwide, population-based study to investigate the incidence of pancreatitis after preeclampsia. Our results revealed that patients with a history of preeclampsia exhibited a 1.68-fold risk of pancreatitis than did those without preeclampsia, after adjusting for demographic characteristics and comorbidities.

Preeclampsia is a common pregnancy-specific disease with potential adverse maternal and neonatal outcomes affecting 3%-5% of all pregnancies [4]. Preeclampsia is an obstetric emergency; in the United States, preeclampsia/eclampsia is one of the four leading causes of death among pregnant women, along with bleeding, cardiovascular disease, and thromboembolism [22–24]. Women with preeclampsia are more likely to suffer from systemic diseases in the future, including hypertension, coronary heart disease, stroke, diabetes, and renal disease [25, 26]. Our study found that women with preeclampsia are more likely to develop comorbidities such as gallstones, diabetes, hyperlipidemia, hypertension, and hepatitis B and C infection than pregnant women without preeclampsia (Table 1), which is consistent with some existing studies [27–30]. In fact, these comorbidities are also risk factors for pancreatitis [31–34]. Pancreatitis may affect surrounding tissues or may cause dysfunction of the distal organ system. A small proportion of patients may have pancreatic necrosis, inflammation of surrounding tissues, and organ failure [35, 36]. The pathophysiology of pancreatitis has not been fully elucidated [37, 38]. The most common factors associated with acute pancreatitis are gallstones (35%–75%) and alcohol consumption (25%–35%), followed by idiopathic causes (10%–20%), hypertriglyceridemia (1%–4%), endoscopic retrograde cholangiopancreatography, and drugs (1.4%–2%) [31–34, 39]. Preeclampsia and pancreatitis seem to have common risk factors, including diabetes, hyperlipidemia, and hepatitis [8, 29–34, 39, 40].

The overall incidence of pancreatitis in the preeclampsia cohort was higher than that in the control cohort, even after adjusting for comorbidities associated with pancreatitis and even when only preeclampsia patients without comorbidities were considered (Table 2). Compared with the control cohort, the incidence of pancreatitis in patients with preeclampsia increased with age, especially in the 26–35 age group; this finding is consistent with studies indicating that advanced maternal age is prone to preeclampsia or pancreatitis [41, 42].

The present study revealed that preeclampsia was an independent risk factor for pancreatitis. However, specific diseases, such as alcohol-related diseases, gallstones, or diabetes, were more influential than preeclampsia. Preeclampsia patients with a single specific comorbidity had a higher risk of pancreatitis than did nonpreeclampsia patients with that comorbidity (aHRs = 1.78–1.89) (Table 3), but the combined effects of preeclampsia and the specific comorbidity further increased the risk of pancreatitis. The risk was especially high for preeclampsia combined with alcohol-related diseases, as well as with hepatitis C, diabetes mellitus, and biliary stone.

The prevalence of pancreatitis in pregnancy is low and ranges between 1 in 1000 and 1 in 3000 deliveries [43], but maternal mortality with severe pancreatitis is high [44]. The incidence of fetal distress and fetal loss increases with the severity of pancreatitis [45]. Fortunately, early diagnosis of pancreatitis during pregnancy and improved maternal–infant intensive care has led to a declined in maternal and infant mortality rates [44]. The most common causes of acute pancreatitis in pregnancy are gallstones (67%–100% of pregnancy cases) [43], alcoholism, and hypertriglyceridemia [44]. Other causes include idiopathic pancreatitis, gestational hypertension, drug-induced pancreatitis, traumatic pancreatitis, and inherited diseases [42, 44, 46].

This study explored the relationship between preeclampsia and pancreatitis. Although no direct link was seen between preeclampsia and the pathology of pancreatitis, preeclampsia has been shown to cause global vascular endothelial dysfunction, which can lead to pancreatitis [7]. The pathophysiology of preeclampsia likely involves both maternal and placental factors, such as abnormalities in the uterine and placental circulations. The resultant ischemic placenta seems to introduce complex factors into the maternal circulation; this leads to maternal vascular endothelial dysfunction and eventually gives rise to the clinical manifestations of preeclampsia [4, 47–50]. Preeclampsia is associated with microvascular abnormalities, which may involve the cerebral, placental, hepatic, renal, and visceral circulation. Thus, the pancreatic vasculature may also get affected and cause acute pancreatitis, which leads to organized pancreatic necrosis [20]. Ramin et al. ^43^ investigated 9 cases of pancreatitis related to preeclampsia and found microthrombosis, intravascular coagulation, and vasculitis during preeclampsia, probably resulting in neurological, renal, hepatic, and placental diseases [43]. Another study reported that preeclampsia can cause acute edematous pancreatitis likely related to microvascular abnormalities and visceral ischemia [51]. Taken together, these findings indicate that maternal vascular endothelial dysfunction in patients with preeclampsia may increase pancreatic vascular system damage, eventually leading to pancreatitis. Further research is warranted to elucidate underlying biological mechanisms.

The difference in the cumulative incidence between patients with preeclampsia and control cohorts increased over time (Figure 1), indicating that the risk of pancreatitis in patients with a history of preeclampsia is long lasting. Because other comorbidities, such as alcoholism, gallstones, hypertension, dyslipidemia, diabetes, and overweight, further increase the risk of pancreatitis, both preeclampsia and comorbidities should be identified as soon as possible and treated effectively to reduce the patient's long-term morbidity and mortality.

## 5. Limitations

The strength of our research was its population-based design. However, the use of an observational database (i.e., NHIRD) has some inherent limitations.

First, the diagnostic accuracy was based on administrative data, making potential misjudging of preeclampsia and pancreatitis results inevitable. However, the Bureau of National Health Insurance randomly cross-checks medical records in all medical institutions to reduce error codes and misclassification bias. We identified hypertensive disorders during pregnancy and other comorbidities by using ICD-9-CM codes. In Taiwan, since the implementation of the National Health Insurance Program, prenatal care has been very well delivered [52]. The program includes 10 routine antenatal checkups, which involve blood pressure measurement and urinary protein tests, allowing accurate and timely diagnosis of preeclampsia by obstetricians. Although we could not calculate the validity of diagnostic codes for hypertensive disorders in pregnancy, the high validity of the diagnostic codes of the NHIRD has been reported [53, 54]. In addition, we used univariable and multivariable Cox proportional hazards models to assess the risk of pancreatitis and expressed them as HRs. Multivariable models were adjusted for age and comorbidities, including the exclusion of only hypertensive patients. The diagnosis of pancreatitis is typically based on clear guidelines involving characteristic symptoms and signs, blood biochemistry results, and imaging findings [55] and is thus not prone to error.

Second, the NHIRD does not include details of high alcohol consumption, body mass index, tobacco use, or other lifestyle-related factors that may be potential confounders. We tried to reduce these confounders by, for example, including alcohol-related diseases as a proxy of alcohol consumption and metabolic syndrome parameters (such as hyperlipidemia and diabetes) as an alternative indicator of body mass index. In general, the smoking rate among Taiwanese women is low, being <2.5% among pregnant women [56]. Moreover, such a large population-based study may have neutralized this effect.

## 6. Conclusions

Compared with patients without preeclampsia, the risk of pancreatitis in patients with preeclampsia was found to be significantly high even after adjusting for certain demographic characteristics and comorbidities. Preeclampsia is an independent risk factor for pancreatitis, and the risk is further increased when accompanied by alcohol-related diseases, gallstones, diabetes, hepatitis C infection, or age of 26–35 years. Thus, timely identification and effective management of preeclampsia and the aforementioned comorbidities can reduce the risk of pancreatitis and the associated morbidity and mortality.

## Figures and Tables

**Table 1:** Demographic characteristics and comorbidities in patients with and without preeclampsia.

Characteristics	Preeclampsia		*p* value
	No	Yes	
	(*N* = 69052)	(*N* = 17263)	
Age stratified			0.99
18-25	8544 (12.4)	2136 (12.4)	
26-35	44216 (64.0)	11054 (64.0)	
36-45	16292 (23.6)	4073 (23.6)	
Age, *mean* ± *SD*^†^	31.0 ± 5.22	31.2 ± 5.19	0.01
Comorbidity			
Alcohol-related disease	64 (0.09)	16 (0.09)	0.99
Biliary stone	722 (1.05)	300 (1.74)	<0.001
Diabetes mellitus	358 (0.52)	866 (5.02)	<0.001
Hyperlipidemia	236 (0.34)	312 (1.81)	<0.001
Hypertension	293 (0.42)	664 (3.85)	<0.001
Hepatitis B	385 (0.56)	127 (0.74)	0.006
Hepatitis C	103 (0.15)	46 (0.27)	<0.001

Chi-square test, ^†^*t*-test.

**Table 2:** Incidence densities of the pancreatitis hazard ratio in women with and without preeclampsia stratified by age and presence of comorbidity.

Variables	Preeclampsia						Crude HR^∗^ (95% CI)	Adjusted HR^†^ (95% CI)
	No			Yes				
	Event	PY	Rate^#^	Event	PY	Rate^#^		
All	113	485211	2.33	52	121327	4.29	1.84 (1.32, 2.55)^∗∗∗^	1.68 (1.19, 2.36)^∗∗^
Stratify age								
≤25	19	66230	2.87	3	16945	1.77	0.61 (0.18, 2.08)	0.58 (0.17, 1.99)
26-35	57	311988	1.83	34	77967	4.36	2.39 (1.56, 3.65)^∗∗∗^	2.22 (1.43, 3.44)^∗∗∗^
36-45	37	106993	3.46	15	26416	5.68	1.63 (0.90, 2.97)	1.40 (0.74, 2.64)
Comorbidity^‡^								
No	99	469860	2.11	41	106185	3.86	1.83 (1.27, 2.63)^∗∗^	1.83 (1.27, 2.63)^∗∗^
Yes	14	15351	9.12	11	15143	7.26	0.80 (0.36, 1.75)	0.80 (0.36, 1.76)

Rate^#^: incidence rate per 10000 person-years; crude HR^∗^: relative hazard ratio; adjusted HR^†^: multivariable analysis including age, alcohol-related diseases, biliary stone, diabetes mellitus, hyperlipidemia, hypertension, hepatitis B, and hepatitis C; comorbidity^‡^: patients with any one of the comorbidities alcohol-related diseases, biliary stone, diabetes mellitus, hyperlipidemia, hypertension, hepatitis B, and hepatitis C were classified as the comorbidity group. ^∗^*p* < 0.05, ^∗∗^*p* < 0.01, ^∗∗∗^*p* < 0.001.

**Table 3:** Cox proportional model to measure the pancreatitis hazard ratio in patients with or without comorbidities.

Variables					Adjusted HR^†^
		Event	PY	Rate^†^	(95% CI)
Preeclampsia	Alcohol-related disease				
-	-	113	484731	2.33	1 (reference)
-	+	0	480	0.00	—
+	-	51	121225	4.21	1.80 (1.29, 2.50)^∗∗∗^
+	+	1	102	97.7	43.4 (6.06, 311.3)^∗∗∗^
Preeclampsia	Biliary stone				
-	-	109	482191	2.26	1(reference)
-	+	4	3020	13.2	5.45 (2.01, 14.8)^∗∗∗^
+	-	46	114489	4.02	1.78 (1.26, 2.51)^∗∗^
+	+	6	6838	8.77	3.63 (1.59, 8.27)^∗∗^
Preeclampsia	Diabetes mellitus				
-	-	104	479566	2.17	1(reference)
-	+	9	5645	15.9	7.13 (3.61, 14.1)^∗∗∗^
+	-	49	119078	4.11	1.89 (1.35, 2.66)^∗∗∗^
+	+	3	2249	13.3	5.89 (1.87,18.6)^∗∗∗^
Preeclampsia	Hyperlipidemia				
-	-	112	483311	2.32	1 (reference)
-	+	1	1900	5.26	2.12 (0.30, 15.2)
+	-	51	118648	4.30	1.85 (1.33, 2.57)^∗∗∗^
+	+	1	2679	3.73	1.51 (0.21, 10.9)
Preeclampsia	Hypertension				
-	-	113	482869	2.34	1(reference)
-	+	0	2341	0.00	—
+	-	51	116693	4.37	1.86 (1.34, 2.59)^∗∗∗^
+	+	1	4635	2.16	0.91 (0.13, 6.52)
Preeclampsia	Hepatitis B virus				
-	-	113	482353	2.34	1 (reference)
-	+	0	2858	0.00	—
+	-	51	120381	4.24	1.80 (1.29, 2.51)^∗∗∗^
+	+	1	946	10.6	4.58 (0.64, 32.8)
Preeclampsia	Hepatitis C virus				
-	-	112	484455	2.31	1(reference)
-	+	1	756	13.2	5.55 (0.78, 39.8)
+	-	51	120965	4.22	1.82 (1.30, 2.53)^∗∗∗^
+	+	1	363	27.6	11.y (1.54, 78.9)^∗^

Rate^†^: incidence rate per 10000 person-years; adjusted HR^†^: multivariable analysis including age; ^∗∗^*p* < 0.01, ^∗∗∗^*p* < 0.001.

## Data Availability

The dataset used in this study is held by the Taiwan Ministry of Health and Welfare (MOHW). The Ministry of Health and Welfare must approve our application to access this data. Any researcher interested in accessing this dataset can apply form to the Ministry of Health and Welfare requesting access. Please contact the staff of MOHW (email: stcarolwu@mohw.gov.tw) for further assistance: Taiwan Ministry of Health and Welfare Address: No.488, Sec. 6, Zhongxiao E. Rd., Nangang Dist., Taipei City 115, Taiwan. Phone: +886-2-8590-6848. All relevant data are within the paper.

## Abbreviations

aHR: Adjusted hazard ratio
CI: Confidence interval
HR: Hazard ratio
ICD-9-CM: International Classification of Diseases, Ninth Revision, Clinical Modification
NHIRD: National Health Insurance Research Databases.

## Additional Points

*Reporting Checklist*. The authors have completed the STROBE reporting checklist. *Data Sharing Statement*. The dataset used in this study is held by the Taiwan Ministry of Health and Welfare (MOHW). The Ministry of Health and Welfare must approve our application to access this data. Any researcher interested in accessing this dataset can apply form to the Ministry of Health and Welfare requesting access. Please contact the staff of MOHW (Email: stcarolwu@mohw.gov.tw) for further assistance: Taiwan Ministry of Health and Welfare Address: No.488, Sec. 6, Zhongxiao E. Rd., Nangang Dist., Taipei City 115, Taiwan. Phone: +886-2-8590-6848. All relevant data are within the paper.
